# Evaluation of Nutritional and Technological Attributes of Whole Wheat Based Bread Fortified with Chia Flour

**DOI:** 10.3390/foods7090135

**Published:** 2018-08-30

**Authors:** Bouchra Sayed-Ahmad, Thierry Talou, Evita Straumite, Martins Sabovics, Zanda Kruma, Zeinab Saad, Akram Hijazi, Othmane Merah

**Affiliations:** 1 Laboratoire de Chimie Agro-Industrielle (LCA), Université de Toulouse, INRA, INPT, 31030 Toulouse, France; thierry.talou@ensiacet.fr (T.T.); othmane.merah@ensiacet.fr (O.M.); 2 Research Platform of Environmental Science, Doctoral School of Science and Technology, Lebanese University, Campus Rafic Hariri, BP 5, 1500 Hadath-Beirut, Lebanon; zsaad2002@yahoo.com (Z.S.); hijazi_akram@hotmail.com (A.H.); 3 Department of Food Technology, Faculty of Food Technology, Latvia University of Life Sciences and Technologies, Riga street 22, LV-3001 Jelgava, Latvia; evita.straumite@llu.lv (E.S.); martins.sabovics@llu.lv (M.S.); zanda.kruma@llu.lv (Z.K.); 4 Département Génie Biologique, IUT A, Université Paul Sabatier, 32000 Auch, France

**Keywords:** chia seed, chia cake, whole wheat bread, antioxidant activity, bread quality

## Abstract

The aim of this study was to investigate and compare the effect of wheat bread fortification with varied levels (2%, 4%, and 6%) of chia seed powder (full fat) and cakes (defatted, residue after oil extraction). Chia flour was added to whole wheat bread rich in vital wheat gluten for the first time. The breadcrumbs were assessed for their antioxidant activity, nutritional content, textural properties, color, and sensory profiles. The addition of chia seed powder, particularly in high levels, was more effective in improving antioxidant activity compared to bread fortified with chia cakes. Bread supplementation with chia flour improves its nutritional value, especially in the case of chia cakes. A higher moisture content and lower hardness were observed after bread fortification, the influence was more evident with the defatted cake than with seed powder. Fortification with chia flour led to darker breads without significantly affecting their global acceptability. However, the fortified bread showed better values than control in terms of sensory profile. These results suggest that the addition of chia seed powder and defatted cake can enhance the overall whole wheat bread quality. Our results also highlight that bread making could be an unconventional alternative for the exploitation of defatted chia seed.

## 1. Introduction

Food technologists are always working forward to ensure the perfect food formulations which please consumer’s growing requests for more natural, functional food with multiple health benefits [1]. Cereal flours, mainly wheat and maize flour, are the typical fortified food worldwide. The aim of wheat flour fortification is to increase the nutritional value as well as the sensory characteristics of produced bread or other baking products [2]. Bread with refined white flour is generally preferred by consumers than that made from whole grain. This can be attributed to the presence of bran in the whole wheat flour products which make their textural properties less attractive. However, the daily consumption of whole grain bread is recommended for a better lifestyle diet [3]. It has been previously proven that the inclusion of new ingredients to whole wheat dough formulations can enhance bread quality and thus consumer’s acceptability [4]. In this regard, vital gluten, which is the insoluble protein portion obtained from wheat flour, can be used in whole wheat bread formulations [5]. Vital gluten addition generally results in an increased protein content, improved dough tolerance, and crumb structure of the final bread [5,6].

*Salvia hispanica*, a *Lamiaceae* species, commonly known as chia, has a long history of use as a food since antiquity. It was a main component in the diet of pre-Colombian people in South and Central America. Chia seeds are a well-known food, their global production is renowned nowadays for their potential health properties. Recently, they have been largely studied because of their increasing popularity and acceptance as a healthy food choice [7]. They are used for cookies and breakfast cereal production, they have also become a popular food supplement in many regions, including Latin America, United States, and Australia. Chia seeds contain 30% vegetable oil, this holds the highest known percentage of α-linolenic fatty acid (up to 67%) [8]. They are also a rich source of protein (19–27%), this content is greater than other typical food crops, including wheat, corn, and rice. They contain 4 to 5% ash, 26 to 41% carbohydrates, and have a high fiber content (18–30%) [9]. Moreover, chia seeds contain a high amount of phenolic compounds and natural antioxidants such as chlorogenic and caffeic acids, quercetin, and kaempferol [10]. Consumption of chia can assure a good functioning digestive system as well as a reduction in cholesterol and glucose levels in blood [11].

After oil extraction, residual meals of chia are reported to have a high content of fiber (19–23%), protein (33.9–39.9%), and antioxidant compounds which are comparable to other oilseeds used in the food industry [12]. In order to obtain suitable meals for food applications, the utilization of the green oil extraction method is a crucial criterion. In this context, single-screw extrusion, which is a solvent free extraction method, appears as a potential alternative. Single-screw pressing, which is a solvent free technique, appears as an ideal choice for oil extraction since it brings out renewable non-toxic raw materials instead of waste [13,14]. Previous studies have investigated the biochemical composition and antioxidant activity of vegetable oils and residual cakes from the extrusion of coriander seeds, they were found to both be compatible with the requirements of the food industry [13,14].

Besides, since the opinion given by the European Food Safety Authority about the safety of chia as a novel food ingredient [15,16,17], a number of studies have investigated the addition of chia seed into commercial product formulations: a better overall quality of white bread was obtained after chia seed and flour addition [16,17]. Moreover, the supplementation of gluten-free bread with chia seed produces products of a better quality [18]. Moreover, a chia diet led to an increase in density-lipoprotein cholesterol and omega-3 fatty acids and a reduction in triacylglycerol levels in rat serum [19]. It has also been observed that the utilization ofs ground seeds could be more beneficial to obtain the potential benefits from bioactive compounds such as fatty acids and fiber [20,21]. However, none of the previous studies have addressed the effect of addition of chia flour on the quality of high gluten whole wheat bread.

The objective of this work was to study the influence of the supplementation of different levels of full fat and defatted chia seed powder on the bread quality. Whole wheat bread with added vital wheat gluten was used. The technological and nutritional characteristics of all formulations were assessed.

## 2. Materials and Methods

### 2.1. Single-Screw Extrusion

Chia seeds were screw pressed using a single-screw (Model OMEGA 20, Villeurbanne, France) press equipped with an 18 cm screw with pitch screws of 1.8 cm, an internal diameter of 1.4 cm, and a channel depth of 0.5 cm. The screw rotation speed was 40 rpm and the feed rate was 0.9 kg/h. The nozzle diameter and the nozzle/screw distance used in the pressing of chia seeds were 5 mm and 3 cm, respectively. The residual cake from the extrusion process was collected after passing through a nozzle and used for further investigation.

### 2.2. Raw Materials and Additives

Whole wheat flour, wheat protein isolate Arise 5000 (GmbH Loryma, Zwingenberg, Germany), sugar, salt, and dry yeast were procured from the local market of Jelgava, Latvia; chia seeds were brought at a local market in Toulouse, France.

### 2.3. Bread Preparation

In order to assess the effect of chia addition on whole wheat bread quality and chemical composition, chia seed powder and cakes were added at 2%, 4%, and 6% of the whole wheat flour amount (Table 1). All ingredients were mixed for 5 ± 1 min at a minimum speed using a dough mixer BEAR Varimixe (Wodschow & Co., Brøndbyvester, Denmark). Dough samples were fermented for 25 min at 36 ± 2 °C temperature. The bread samples were then baked at 200 ± 5 °C for 20 min in a rotating connection oven (Sveba Dahlen, Fristad, Sweden) and then cooled at room temperature (22 ± 2 °C) for 2 h. 

### 2.4. Theoretical Nutritional Value Calculation

The contents of carbohydrates, protein, fat, and fiber of the protein bread samples were determined according to EU Regulation No. 1169/2011 on the provision of food information to consumers using the following converting actors: Carbohydrates (except polyols): 4 kcal·g^−1^; protein: 4 kcal·g^−1^; Fat: 9 kcal·g^−1^, and Fibre: 2 kcal·g^−1^. 

### 2.5. Moisture Content Analysis

The moisture content of the protein bread was determined using standard method ISO 712:2009 [22]. Measurements were made in triplicate. 

### 2.6. Textural Analysis

After cooling for at least 2 h, hardness analyses were performed using a TA-XT plus Texture Analyzer (Stable Micro Systems Ltd., Surrey, UK) equipped with a cylindrical probe of 25 mm in diameter (test speed: 1 mm/s, trigger force: 0.049 N and distance: 4 mm to the bread slice). All values are given as an average of six measurements. 

### 2.7. Crumb Color Analysis

The CIE Lab coordinate of bread samples was determined using Color Tec-PCM/PSM (Accuracy Microsensors Inc., Monroe Ave Pittsford NY, USA) reporting luminosity (L*), redness (a*), and yellowness (b*). Measurements were done at five different points within the crumb region; mean values were reported for each sample.

The total color differences (*ΔE*) were calculated using Minolta equations as follows:

∆𝐿 = (𝐿 − 𝐿_0_); ∆𝑎 = (𝑎 − 𝑎_0_); ∆𝑏 = (𝑏 − 𝑏_0_) (1)

∆𝐸 = √(∆𝐿^2^ + ∆𝑎^2^ + ∆𝑏^2^) (2)

where *L*, *a* and *b*—measured values of protein bread samples with chia flour; *L*_0_, *a*_0_, and *b*_0_—the values of the protein bread (control).

### 2.8. Sensory Evaluation Analysis

The sensory evaluation of bread samples was carried out for consumer acceptance and preference using thirty-one panelists of both sexes aged 20–32 years from students’ control and food expertise, Faculty of Food Technology, Latvia University of Agriculture using a five point Hedonic Scale (1 and 5, representing extreme dislike and extreme like, respectively). Coded samples of the same size were served to participants in identical containers each in a panel cupboard.

### 2.9. Total Phenolic Content (TPC) and Trolox Equivalent Antioxidant Capacity (TEAC) Determination

A mixture solution consisting of ethanol/acetone/water (7/7/6 *v*/*v*/*v*) was used to extract protein bread samples. Ultrasonic extraction was done in an ultrasonic bath YJ5120-1 (Oubo Dental, NY, USA) at 35 KHz for 10 min at 20 ± 1 °C and then centrifuged using a CM-6MT (Elmi Ltd., Riga, Latvia) centrifuge at 3500 rpm for 5 min [13]. A similar process was then used to re-extract the remaining bread and supernatant was combined. A triplicate extraction process was done for each sample. 

TPC of the protein bread extract was estimated by the Folin–Ciocalteu method [23] with some modifications. A 0.5 mL extract was mixed with 2.5 mL of Folin–Ciocalteu reagent (diluted 10 times with water). After 3 min, 2 mL of saturated sodium carbonate (75 g·L^−1^) solution was added to the mixture. The reaction was kept in the dark for 30 min, after which the absorbance was read at 765 nm. Gallic acid was used to construct the standard curve. The results were mean values + standard error of mean and expressed as expressed as Gallic acid equivalents (GAE) 100 g^−1^ dry weight (DW) of the samples. Measurements were made in three biological triplicates for each sample.

The 2,2-diphenyl-1-picrylhydraziyl (DPPH) method [24] with slight modifications was used to determine the antioxidant activity of the extract. A solution of DPPH solution (4 mg DPPH in 100 mL methanol) was prepared and then 3.5 mL of the DPPH solution was mixed with 0.5 mL of extract. Finally, the samples were incubated for 30 min in the dark at room temperature. The scavenging capacity was read spectrophotometrically by monitoring the decrease in absorbance at 517 nm. Using a UV–VIS spectrophotometer (JENWAY 6300, Staffordshire, UK). The radical scavenging activity was expressed as Trolox mM equivalents (TE) 100 g^−1^ dry weight (DW) of the samples. Measurements were made in three different biological triplicates for each sample.

### 2.10. Statistical Analysis

Analysis of variance was conducted using one-way ANOVA and Tukey test by pairwise analysis with significance defined at *p* < 0.05. All experiments were carried out in triplicate (each point of them was a mean of triplicate technical measurements) and the mean values were expressed.

## 3. Results

### 3.1. Nutritional Analysis of Bread

The calculated nutrient content of all enriched breads is given in Table 1. Generally, chia flour addition produced an increase in selected nutritional properties as a function of the increasing rate of chia fortification in the formulations. Samples fortified with chia cakes showed higher carbohydrates, proteins, and fiber contents than those fortified with chia seed powder. The fiber content in bread fortified with 6% of chia cakes (Chic6) was the highest; it increased by 17% compared to the control bread. Our findings are in line with previous works which reported that the fiber content of fortified bread increased by 19% compared to control [9]. Our results are higher than those obtained by Justo et al. (2007), they found that bread supplementation with wheat:soybean:chia:flaxseed (80:10:05:05) resulted in a 1.78% increase in fiber content [25]. It has been reported that a high dietary fiber intake helps to prevent and reduce the risk of several diseases including diabetes, obesity, cardiovascular diseases, and hypertension [26]. A lower fat content was obtained in samples fortified with chia cakes than those fortified with chia seed powder which was expected as our chia seed powder contains more fat than chia cakes (Table 1). The higher energy values of the samples with chia seed powder are also due to their greater oil content. The previous studies also showed that chia fibrous fraction and by-products are a promising ingredient for functional food production including cookies, bars, bread, and others [7,27].

### 3.2. Bread Moisture Content

**Figure 1:**
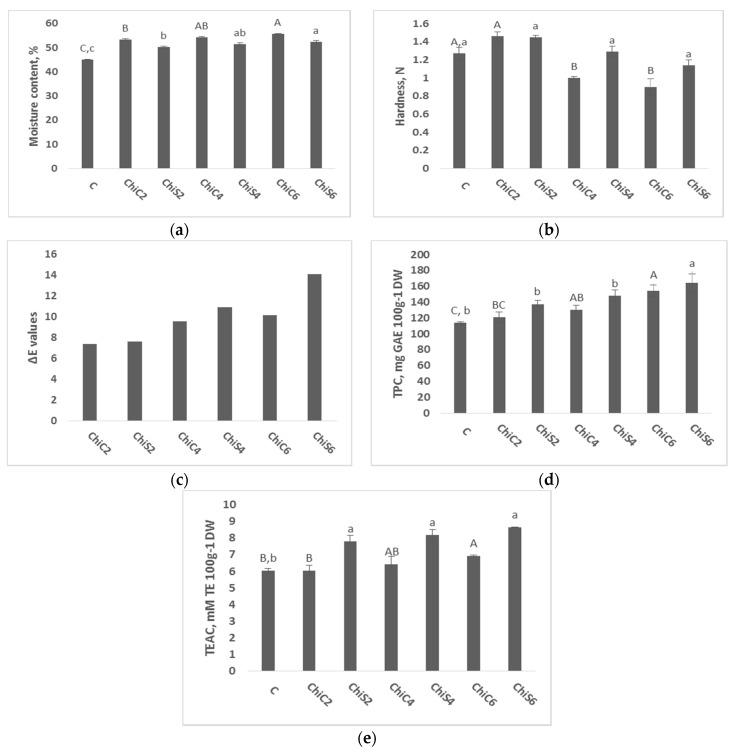
(**a**) Moisture content (%); (**b**) hardness (N); (**c**) Total color difference (Δ*E*); (**d**) Total phenolic content (TPC, expressed as mg GAE 100 g^−1^ DW); (**e**) Trolox equivalent antioxidant capacity (TEAC expressed as mM TE 100 g^−1^ DW) of whole wheat bread samples fortified with chia seed powder and cakes. Column marked with the same subscript letters in each bar chart are not significantly different (*p* > 0.05).

The moisture content significantly augmented by increasing the level of fortification with chia seed powder and cakes (Figure 1a). This can be attributed to the fact that the addition of chia powder in bread causes greater holding of crumb moisture. These results are in line with previous studies where a higher moisture content was also obtained after the addition of chia flour to bread [9,28]. Moreover, the moisture content of fortified bread showed an increase of 23.6% and 16.7% in ChiC6 and ChiS6, respectively, compared to control bread (Figure 1a). The higher moisture content in samples fortified with chia cakes than those fortified with chia seed powder could be due to the higher amount water-soluble fiber in the bread containing chia cakes [29].

### 3.3. Bread Hardness Analysis

Textural properties are an important feature in defining bread quality, softness, and strength characteristics [30]. The hardness of the fortified bread decreased steadily by increasing the level of fortification (Figure 1b), especially in those fortified with chia cakes. Similar results obtained previously reported that bread firmness showed a tendency to decrease after chia addition [11,28]. These results can be attributed to the presence of hydrocolloids (mucilage) in chia seeds [31]. It has been previously proved that the addition of hydrocolloids into bread doughs results in a reduction in their hardness [32]. However, different parameters can affect the final texture of bread including baking conditions (temperature and time) and bread components (fiber, protein, starch) [33,34].

### 3.4. Bread Color Analysis

Values of L* and b* decreased while those of a* and ΔE* significantly increased with the augmentation of chia level in the bread formulation (Table 1 and Figure 1c). Crumb color had changed from brown (control) to darker brown (ChiS6 and ChiC6). Similar results were found in the literature where darker bread was obtained after the addition of chia seeds [9,28]. The color change is logically explained by the presence of the chia pigment itself which is darker than whole wheat flour. The presence of phenolic compounds such as caffeic acid, chlorogenic acid, ferulic acid, p-coumaric acid, and others are responsible for the dark color of chia seed [28]. Bread darkening can also be due to non-enzymatic chemical reactions such as caramelization and Maillard browning reaction between wheat protein with added sugar that produce colored compounds [35].

### 3.5. Bread Sensory Analysis

Consumer’s acceptability is an important parameter for introducing a new product into the market. The result of the sensory analysis of the bread samples containing different levels of chia substitution compared to the control is shown in Table 1. The statistical analysis revealed that there is no significant difference among all bread samples in the overall sensory attributes. The mean sensory scores ranged between 3.4 and 3.7 (Table 1). However, hedonic scores of substituted bread were greater than control bread, samples with added chia flour were generally well-liked as their scores were higher than 3.5. It must be taken into account that the panelists were not familiarized with whole wheat bread with vital gluten. Our results are in agreement with those obtained by Steffolani et al. [18], where no significant differences in the global acceptability were obtained between gluten-free bread fortified with 15% of chia flour and control bread [14].

### 3.6. TPC and TEAC Analysis of Bread

Bread extracts were analyzed for their total phenol content (TPC); results were expressed as Gallic Acid (GAE) per 100 g dry weight (DW). Chia addition had a positive impact on the levels of these phytochemicals in all samples as TPC values showed significant variation with respect to chia seed powder and cakes level (Figure 1d). However, phenol contents varied between samples fortified with chia seed powder and cakes. The TPC of the bread with seed powder was consistently higher than that with cakes across all chia replacement values.

The total antioxidant activities (TEAC) determined by DPPH of whole wheat breads were expressed as mM of Trolox per 100 g dry weight (Figure 1e). Results of the antioxidant assays were in line with the TPC results, they indicated the same trends. TEAC significantly increased in fortified bread in comparison with control bread. Bread fortified with chia seed powder showed also higher activity than those fortified with chia cakes at different levels. However, these results were expected as chia seeds are considered as a potential source of antioxidants due to the presence of polyphenols including chlorogenic and caffeic acids, myricetin, quercetin, and kaempferol [36]. Moreover, it was reported that residual cakes, after oil extraction, are considered to be a good source of natural antioxidants [37]. It has also been found that bread fortification with pseudo-cereals increased the antioxidant potential of obtained bread, which is in line with our results [38,39]. El-Demery et al. (2015), obtained higher antioxidant activity in bread fortified with full fat flaxseed than that fortified with defatted flaxseed [20].

## 4. Conclusions

This study has shown that chia seeds can be used as a raw material to improve the overall quality of whole wheat bread containing a high percentage of vital gluten. These findings may help in the development of enhanced functional commercial whole wheat bread and for the effective valorization of chia seeds and the related residues. This species could be utilized for a variety of food uses as well as being valuable in bio-refining processes thus using several products and residues.

## Figures and Tables

**Table 1:** Crumb color analysis, nutritional, energy values, and mean Hedonic values of different bread samples.

	Color			Nutrients, g 100 g^−1^				Energy Value, 100 g^−1^	Sensory Evaluation
Bread Samples	L*	a*	b*	Carbohydrates	Protein	Fiber	Fat	kcal	Mean Hedonic Values
**Control (C)**	61.08 ^a^ ± 2.06	0.47 ^d^ ± 0.69	20.32 ^a^ ± 1.96	25.59	21.37	4.96	0.97	210.49	3.39
**2% of chia seed powder (ChiS2)**	54.00 ^b^ ± 1.73	1.54 ^c^ ± 0.04	17.68 ^ab^ ± 0.79	25.75	21.63	5.07	1.29	223.19	3.73
**4% of chia seed powder (ChiS4)**	51.88 ^b^ ± 0.09	2.18 ^c^ ± 0.21	14.65 ^b^ ± 0.01	25.92	21.61	5.18	1.61	235.77	3.55
**6% of chia seed powder (ChiS6)**	48.84 ^b^ ± 1.89	4.30 ^b^ ± 0.18	14.48 ^b^ ± 0.07	26.08	21.60	5.29	1.92	248.41	3.68
**2% of chia cakes (ChiC2)**	54.47 ^b^ ± 0.32	3.84 ^b^ ± 0.41	20.33 ^a^ ± 0.91	26.04	21.74	5.20	1.01	221.45	3.68
**4% of chia cakes (ChiC4)**	52.55 ^b^ ± 0.20	4.67 ^b^ ± 0.01	18.99 ^a^ ± 1.28	26.49	21.83	5.43	1.06	232.41	3.65
**6% of chia cakes (ChiC6)**	52.69 ^b^ ± 1.30	5.99 ^a^ ± 0.44	18.56 ^a^ ± 0.35	26.94	21.92	5.66	1.11	243.37	3.58

L*: luminosity, a*: redness, b*: yellowness. Values marked with the same subscript letters in rows are not significantly different (*p* > 0.05).
